# Supplementary material for: Association between vaccination rates and COVID-19 health outcomes in the United States: a population-level statistical analysis
Source: BMC Public Health. 2024 Jan 18;24:220. doi: 10.1186/s12889-024-17790-w (PMC10797940; doi:10.1186/s12889-024-17790-w)
Supplement: Supplementary file 1 — Supplementary Material 1: Supplementary Data. Supplementary Methods. Supplementary Results [file 12889_2024_17790_MOESM1_ESM.docx]

Supplementary Materials for

**Association between vaccination rates and COVID-19 health outcomes in the United States: a population-level statistical analysis**

Hongru Du MS^1,2, *^, Samee Saiyed MSE^1,2^, and Lauren M. Gardner PhD^1,2,3^

^1^Center for Systems Science and Engineering, Johns Hopkins University, Baltimore, MD 21218, USA.

^2^Department of Civil and Systems Engineering, Johns Hopkins University, Baltimore, MD 21218, USA.

^3^Department of Epidemiology, Johns Hopkins Bloomberg School of Public Health, Baltimore, MD, 21205, USA.

*Corresponding author. Johns Hopkins University, 3400 N. Charles Street, Shaffer 4, Baltimore, MD, 21218, USA. E-mail address: hdu9@jhu.edu (Hongru Du)

Table of Contents

[1. Supplementary Data 2](#_Toc143437339)

[*1.1 Preprocessing of genomic data* 2](#_Toc143437340)

[*1.2 Variables description* 3](#_Toc143437341)

[*1.3 Preprocessing of mobility data* 7](#_Toc143437342)

[2. Supplementary Methods 8](#_Toc143437343)

[*2.1 Static variables selection* 8](#_Toc143437344)

[*2.2 Dynamic variables selection* 9](#_Toc143437345)

[*2.3 Robustness check of vaccination data* 10](#_Toc143437346)

[*2.4 Sensitivity analysis of prior window length for previous infection* 11](#_Toc143437347)

[*2.5 Sensitivity analysis of lags for previous infection* 12](#_Toc143437348)

[*2.6 GAMs with reported case-incidence rate (RCIR) as the outcome variable* 13](#_Toc143437349)

[3. Supplementary Results 14](#_Toc143437350)

[*3.1 Models evaluation for GAMs with RCHR as outcome variable* 14](#_Toc143437351)

[*3.2 Models evaluation for GAMs with RCIR as outcome variable* 18](#_Toc143437352)

**1. Supplementary Data**

*1.1 Preprocessing of genomic data*

All genomic data were collected from GISAID [1] on October 27^th^, 2022. GISAID is a publicly accessible repository of dataset that sharing of genomic data on various pathogens, including influenza and COVID-19. We analyzed the available set of sequences, to determine the proportion of each variant theoretically in circulation. Specifically, we calculated the proportion of each variant for each week in each state from March 1^st^, 2021, to March 1^st^, 2022. To identify the most dominant variant for each state-week pair during the analyzed period, we labeled the state-week pairs based on the variant with the highest proportion. This enables us to track the dominant variant in each state and cluster the state-week pairs based on the most dominant variant. The assignment of state-week pairs is shown in Appendix figure S1 below:


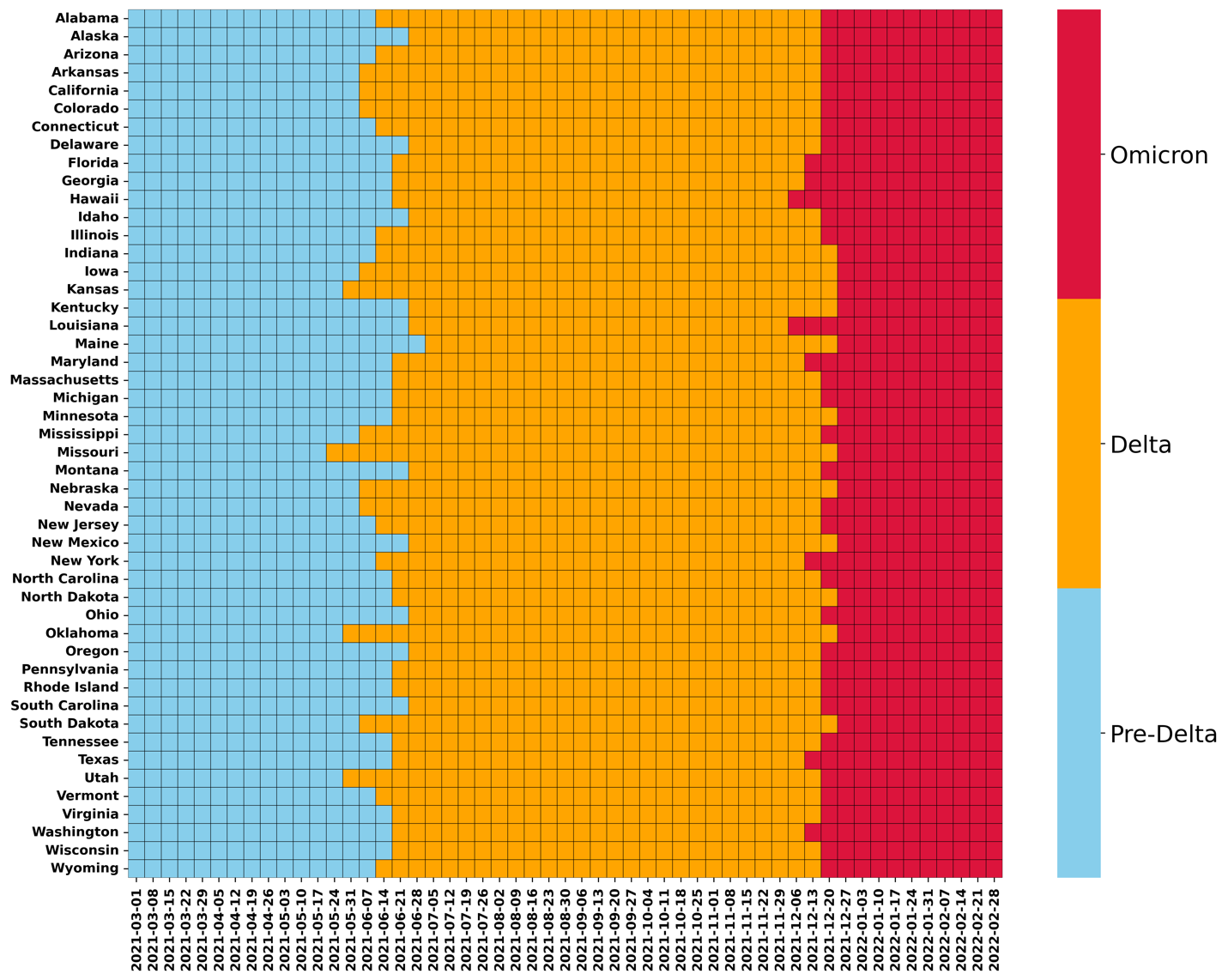


Appendix figure S1: State-week group assignment based on the dominant variant. The x-axis represents each week, the y-axis represents each state, and the color represents the assignment of each state-week pair.

*1.2 Variables description*

***Outcome variable***

Case-hospitalization risk: In this study we used case-hospitalization risk (${CHR}_{i}^{t}$) as the outcome variable of interest for our model. COVID-19 case-hospitalization risk represent both the severity of COVID-19 disease at an individual level and the burden it places on the healthcare system. Case-hospitalization risk (C${HR}_{i}^{t}$) for each state $i$ and week $t$ is defined as follow:

${CHR}_{i}^{t}= \frac{H_{i}^{t}}{C_{i}^{t-1}}$,

where $H_{i}^{t}$ is the number of hospitalizations for state $i$ and week $t$ and $C_{i}^{t-1}$ is the number of confirmed cases for state $i$ and week $t-1$. We applied a one-week lag between reported confirmed cases and hospitalizations to account for the time between symptom onset and hospital admission [2]. Weekly state-level ${CHR}_{i}^{t}$ was treated as the outcome variable in this analysis and served as an indicator for the burden of COVID-19 risk for a given time and location.

***Dynamic Covariates***

Vaccination rate: In this analysis, the weekly cumulative COVID-19 vaccination rate is the primary variable under examination, as we hypothesize it to be a critical determinant in protecting populations against severe COVID-19 disease. The completed primary series rate was chosen over the partial vaccination rate because it represents the recommended dosage by the U.S. CDC. To address the fact that vaccine eligibility was not available to all U.S. adults until April 19^th^, 2021, we also ran the model using the partial vaccination rate for the pre-Delta waves, and the results are consistent with the completed primary series rate (results are presented in Appendix section 2.3). As booster shots became widely available during the Omicron wave, we also include the booster vaccination rate as a covariate to investigate its potential impact on COVID-19 health outcomes. Due to errors and anomalies in the data, the vaccination data excludes West Virginia and New Hampshire, while the booster vaccination data excludes West Virginia, New Hampshire, and North Carolina. As a result, West Virginia and New Hampshire are excluded from all analyses, and North Carolina is excluded from the booster analysis for the Omicron wave.

Activity-related engagement level: We adapted multiple mobility-derived metrics from a previous study [3] to represent destination-specific travel behaviors and activity-related engagement levels for specific types of activities, namely gym, university, and physician visits. Specifically, the engagement levels represent the weekly number of visits to a given destination of interest per person per week. This variable allows us to compare the relative frequency of visits to each point of interest across states and to investigate their potential impact on COVID-19 health outcomes. The metrics were generated based on anonymized mobility data from Safegraph [4], which tracks the number of visits to different types of destinations for a sample of the population at the census tract level in the U.S. Examples of destinations include full-service restaurants, gyms, and grocery stores. The original Safegraph dataset includes over 20 destination categories; thus, to reduce the complexity of the model we identified a smaller representative set of destinations to include as input in the final model. This was accomplished by first organizing the destination categories into six distinct destination groups based on the first two digits of the NAICS code [5], namely Retail Trade (44-45), Education Services (61), Healthcare and Social Assistance (62), Arts, Entertainment, and Recreation (71), Accommodation and Food Services (72), and Other Services (81). From each group, we selected one destination category as the representative variable for the group based on the correlations between other variables within the group (Details are documented in Appendix section 1.4). Subsequently, we conducted a model selection process to identify the most appropriate subset of mobility variables from these six to be included in the final model based on concurvity and significane level (details are documented in Appendix section 2.2).

Previous infection rates: Several studies have demonstrated the effectiveness of previous infections against reinfections and severe COVID-19 outcomes. Studies have illustrated that individuals retain a substantial level of natural immunity for six months after infection [6–8]. To account for the role of acquired immunity from previous infection in protecting from severe disease upon reinfection, we generate a variable to represent the total population infected and recovered within a recent window, i.e., the total infections reported between weeks (t-16) and (t-4). This designated window helps us consider the time period required to recover and build up immunity [9] by the time period t at which the hospitalizations are modeled, while keeping a short enough window that the immunity has not waned. This specific prior infections variable (${PI}_{i}^{t}$), requires multiple parameters, namely the length of the interval that infections are summed over and the start and end period of the window. To identify the best window and evaluate the sensitivity of our analysis to the chosen window length, start and end time, we conducted a sensitivity analysis. The time window with the largest deviance explained in the GAMs was selected for the final model, which was a three-month window ranging from 4 to 16 weeks prior to time t. Additional details of this sensitivity analysis and the results are included in Appendix section 2.4. The mathematical formulation of this metric is defined as follows:

$${PI}_{i}^{t} = \frac{\sum_{j:t-16}^{t-4} C_{i}^{j}}{{pop}_{i}}$$

where ${PI}_{i}^{t}$ represents cumulative infection rate for state $i$ from 16 to 4 weeks prior of week t, $C_{i}^{j}$ is the weekly confirmed cases for state $i$ at week $j$, and ${pop}_{i}$ is the population for state $i$. The sum in the numerator defines the summation of $C_{i}^{j}$ for the $t-16$ to $t-4$ weeks prior to t.

Government policy: The stringency and timing of implementing government policies to mitigate the impacts of COVID-19, such as school closure, cancellation of public events, and international travel controls, are associated with different measures of epidemic severity [10]. We selected the government response index from Oxford Coronavirus Government Response Tracker (OxCGRT) [11] as our indicator for government policy. The index tracks the diversity of government responses across various policies, ranging from containment measures and closures to healthcare systems, vaccination strategies, and economic policies. This index reflects the government’s response level with a number ranging from 0 to 100, the larger the number, the more substantial the response. It is available for all 50 states in the U.S. at a weekly timescale for the entire period of analysis.

Weekly testing rate: The weekly testing rates were included in the model as a potential confounding factor for multiple reasons. Firstly, it represents a proxy input feature to capture the level of healthcare infrastructure available to a population. Second, it directly impacts the case-hospitalization risk through the denominator (i.e., total reported cases), as the number of reported cases in a region is a direct function of local testing availability, thus increased testing will lead to higher reported case rates, and lower case-hospitalization risk. For example, in two locations with the same true case-hospitalization risk (e.g., the likelihood of a COVID-19 infection needing admittance is equal), a location with twice as much testing will detect more cases, and therefore appear to have a lower case-hospitalization risk. Third, increased testing can lead to more cases being identified, and thus impact people’s awareness and behavior during an outbreak. For these reasons testing rate is included as a potential confounding factor in our model. We normalized the raw weekly total testing count by population to get the weekly testing rate.


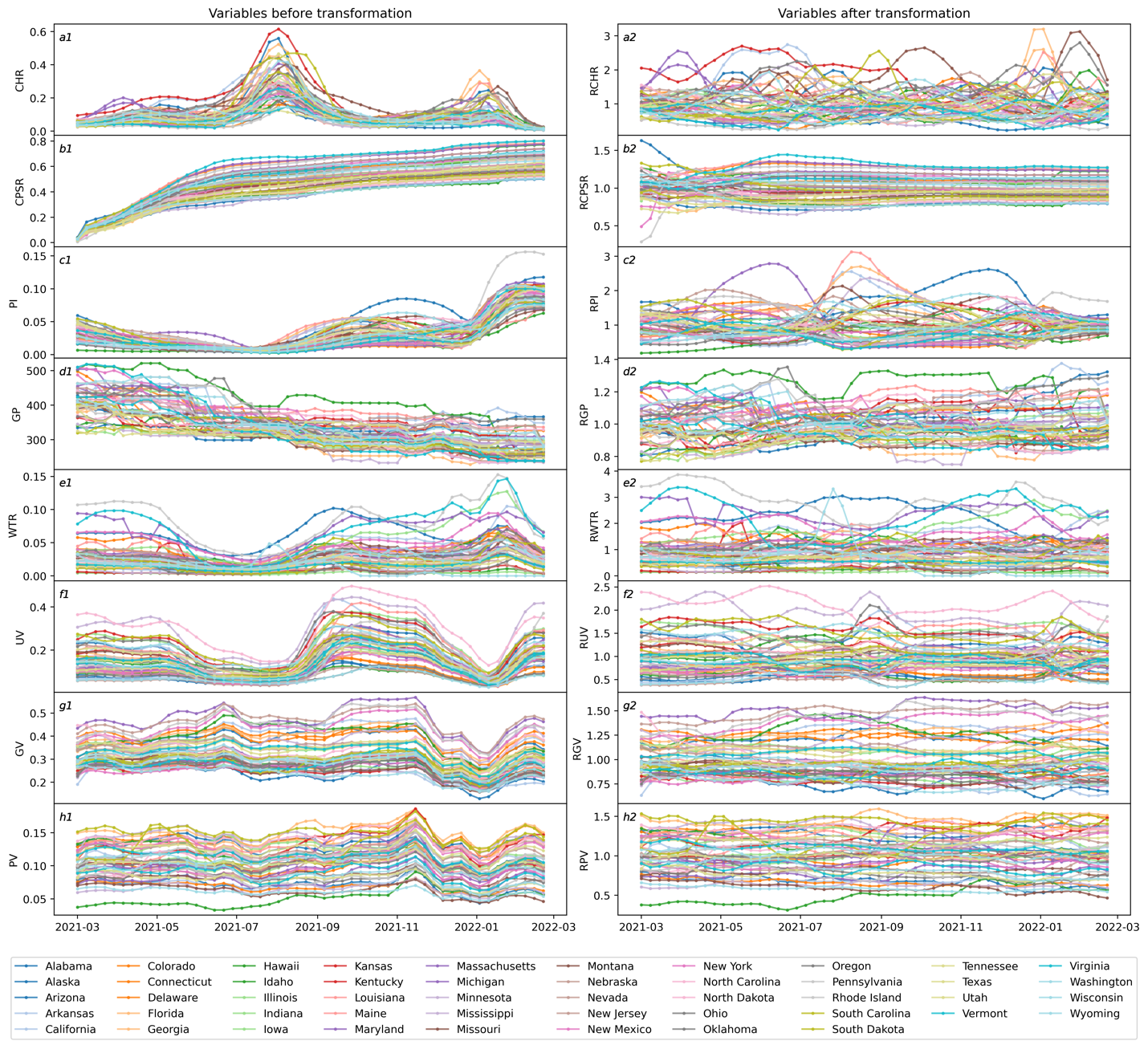


Appendix figure S2: Visualization of dynamic variales before variable transformation (a1 to h1) and after variable transformation (a2 to h2). For the y-axis label, the abbreviations signify the following variables: CHR: Case-hospitalization risk, CPSR: Completed primary series rate, PI: Previous infection, GR: Government policy measure, WTR: Weekly testing rate, UV: University visits, GV: Gym visits, PV: Physician visits.

**Static** ***Covariates***

Appendix table S1: Full list of static variables.

| Variable name | Variable description | source |
| --- | --- | --- |
| Static variables | | |
| Black proportion | The proportion of the population identified as Blacks. | [12] |
| White proportion | The proportion of the population identified as Whites. | [12] |
| Native Hawaiian/Other Pacific Islander Proportion | The proportion of the population identified as Native Hawaiian/Other Pacific Islanders. | [12] |
| Native American/Alaska Native proportion | The proportion of the population identified as Native American/Alaska Natives. | [12] |
| Asian proportion | The proportion of the population identified as Native American/Alaska Natives. | [12] |
| Multiple Races proportion | The proportion of the population identified as two or more races. | [12] |
| Medicaid spending | Total Medicaid spending in kilo for each state normalized by the population. | [13] |
| Healthcare spending | Total Healthcare spending in kilo for each state normalized by the population. | [14] |
| Poverty rate | Percentage of population living below poverty line. | [15] |
| Social Vulnerability Index | The Social Vulnerability Index utilizes data from the U.S. Census to assess the relative level of social vulnerability in each census tract. By analyzing 14 social factors, the SVI categorizes tracts into four closely interrelated themes and then aggregates them as a single indicator of social vulnerability. | [16] |
| HAQI | IHME’s healthcare access and quality index. | [17] |
| Republican voters | Percentage of a state’s voters who voted for the 2020 Republican presidential candidate. | [18] |
| Adults at high risk | The proportion of the population over 18 years old is at high risk of serious illness if infected with Coronavirus. | [19] |
| Proportion over 65 | Proportion of population age 65 and older. | [19] |

*1.3 Preprocessing of mobility data*

The 21 mobility destination categories from Safegraph were organized into six distinct industry groups based on the NAICS code. The relevant groups per the NAICS code are Retail Trade (44-45), Education Services (61), Healthcare and Social Assistance (62), Arts, Entertainment, and Recreation (71), Accommodation and Food Services (72), and Other Services (81) [5]. The details of generating visits to each POI are documented in a previous publication [3].

Appendix table S2: Description of each industry group and the corresponding destination categories.

| Retail Trade (44-45) | Education Services (61) | Healthcare and Social Assistance (62) | Arts, Entertainment, and Recreation (71) | Accommodation and Food Services (72) | Other Services (81) |
| --- | --- | --- | --- | --- | --- |
| Automotive Store (441310) | Elementary School (611110) | Office of Physician (621111) | Parks (712190) | Hotels (721110) | Religious Organizations (813100) |
| Hardware Store (444130) | University (611310) | Child Day Care (624410) | Gym (713940) | Full-Service Restaurant (722511) |  |
| Grocery Store (445110) |  |  |  | Cafes, Snacks, Bars (722515) |  |
| Convenience Store (445120) |  |  |  | Limited-Service Restaurant (722513) |  |
| Pharmacies (446110) |  |  |  |  |  |
| Gas Station (447110) |  |  |  |  |  |
| Sporting Goods Store (451110) |  |  |  |  |  |
| Department Store (452210) |  |  |  |  |  |
| Other General Store (452319) |  |  |  |  |  |
| Used Merchandise Store (453310) |  |  |  |  |  |

To reduce the complexity of the model, we selected one destination category as the representative variable for each industry group. For the industry groups with more than 3 destination categories, we conducted a Pearson’s correlation analysis and selected the variable that had the highest correlation to the other destination categories in each group. This method selected Gas Stations and Full-Service Restaurant from the Retail Trade (44-45) and Accommodation and Food Services (72) as the representative variable for these industry groups. For the Educational Services (61) and Healthcare and Social Assistance (62) groups, we selected University and Office of Physician as the representative variables based on studies that indicated SARS-CoV-2 infection severity is lower in adolescents than adults [20]. For the Arts, Entertainment, and Recreation (71) industry group, we selected Gym as the representative variable instead of Parks because studies have identified park use to have a minor beneficial effect on COVID-19 transmission compared to other mobility destinations [21]. Religious Organizations was selected from the Other Services (81) industry group because it is the only destination category present.

**2. Supplementary Methods**

*2.1 Static variables selection*

Our decision to include only the Black proportion as a key variable was informed by two critical observations: firstly, other racial groups, except the White and Black, are disproportionately represented across the U.S., as shown in Appendix figure S3 below. Secondly, there exists a high negative correlation (-0.65) between the proportions of Black and White populations across states. This strong relationship allowed us to adequately capture racial demographic variations using just the Black proportion, thus simplifying the model without compromising the integrity of our analysis.
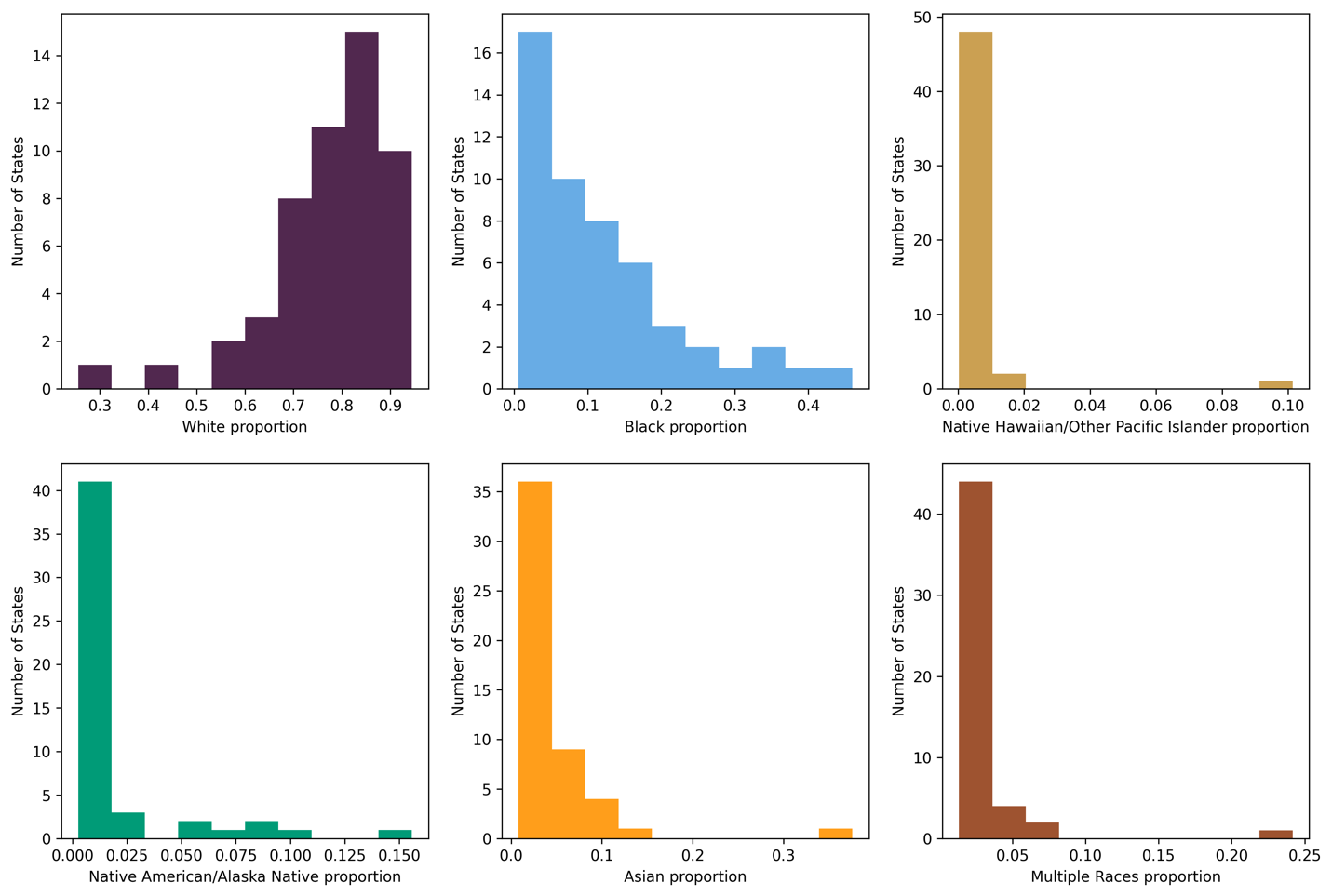


Appendix figure S3. Distribution of these six racial groups across the U.S.

We selected other state-level static variables that were found to have association with COVID-19 health outcomes in a recent study [22]. These variables cover a range of different factors, such as socioeconomic indicators, racial demographics, age, proxy for comorbidities, political factors, and state-level healthcare expenditures. Then, a correlation analysis is performed within static variables to determine the suitable variables to be included in the model. Full list of static variables included in the correlation analysis are listed in Appendix table S1.


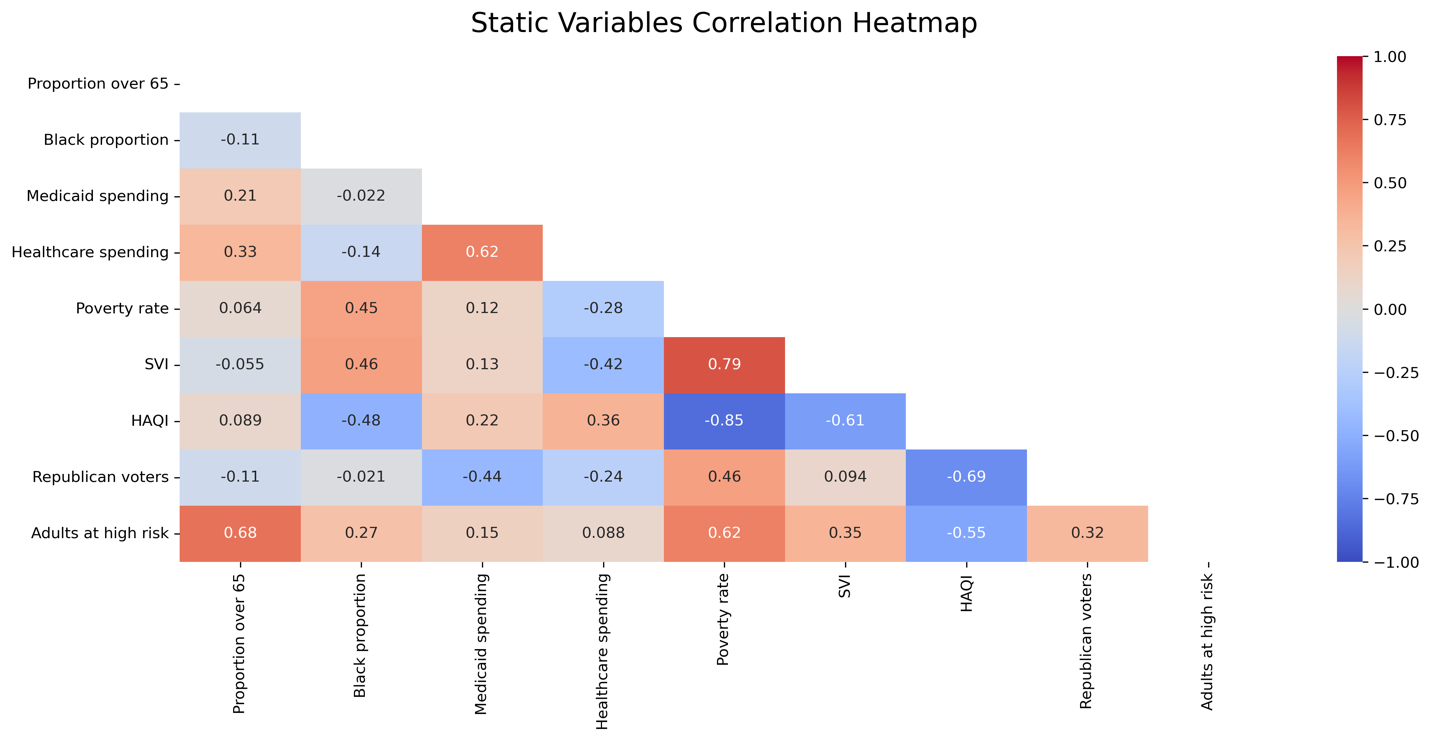


Appendix figure S4. Pearson’s correlation heatmap between each pair of static variables.

Based on the correlation analysis, the set of static variables to be incorporated into the models was determined. We selected the black proportion as a representative variable for the race group and the SVI as a proxy for the vulnerable population. The poverty rate was dropped due to its high correlation with SVI. Additionally, we selected adults at high risk as a control variable for population-level comorbidities and dropped the proportion over 65. We also decided to include Medicaid spending over healthcare spending as the state-level proxy for healthcare expenditures. Lastly, we dropped the HAQI and Republican voters variables due to their high correlation with the completed primary series rate.

*2.2 Dynamic variables selection*

A summary of all the dynamic variables are provided in Appendix table S3 below:

Appendix table S3: Summary dynamic variables.

| Dynamic variables | Mean | St.d. | Min | Max |
| --- | --- | --- | --- | --- |
| Relative case-hospitalization rate | 1 | 0.416 | 0.235 | 3.026 |
| Relative partial vaccination rate | 1 | 0.149 | 0.268 | 1.541 |
| Relative completed primary series rate | 1 | 0.153 | 0.292 | 1.641 |
| Relative booster vaccination rate | 1 | 0.263 | 0.526 | 1.942 |
| Relative previous infection rate (12 weeks) | 1 | 0.409 | 0.154 | 3.068 |
| Relative full-service restaurant visits | 1 | 0.164 | 0.562 | 1.518 |
| Relative gas station visits | 1 | 0.331 | 0.418 | 2.152 |
| Relative religious organization visits | 1 | 0.272 | 0.325 | 1.654 |
| Relative gym visits | 1 | 0.207 | 0.616 | 1.623 |
| Relative university visits | 1 | 0.388 | 0.343 | 2.478 |
| Relative office of physician visits | 1 | 0.248 | 0.322 | 1.560 |
| Relative weekly testing rate | 1 | 0.646 | 0.000 | 3.901 |
| Relative government response index | 1 | 0.109 | 0.736 | 1.375 |

The correlation between each pairs of dynamic variables included in this study are shown in Appendix figure S5 below:

*
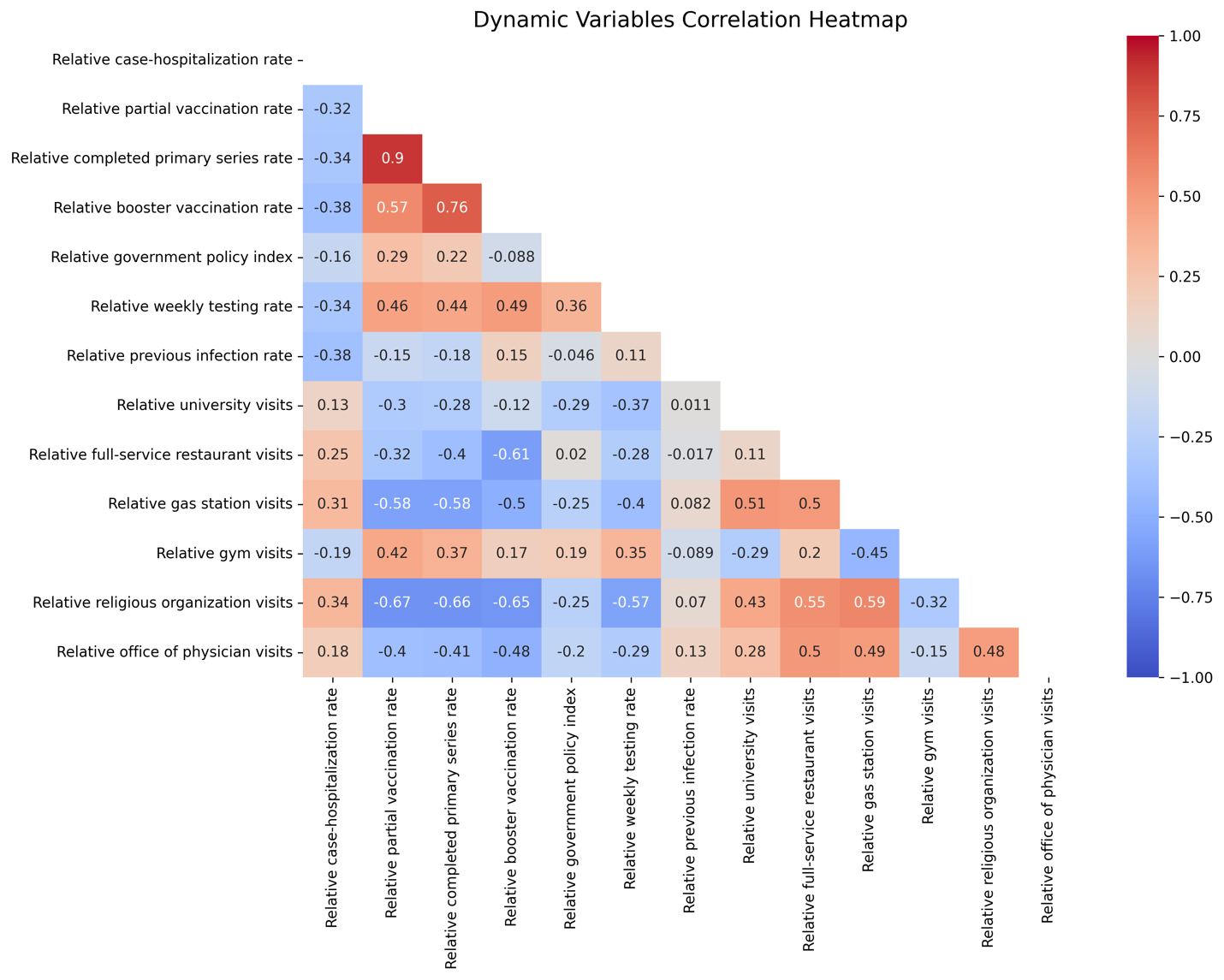
*

Appendix figure S5. Pearson’s correlation heatmap between each pair of dynamic variables.

The GAMs fit outcome variables with smoothed independent variables, allowing the nonlinear relationships between input and output. However, the nonlinear variables smoothing sometimes can result in concurvity issues. Concurvity occurs when some smooth term in a model could be approximated by one or more of the other smooth terms, leading to inaccurate estimates of the effect for given variables. In this section, we conduct model selection to ensure the validity of our model and to detect and mitigate any concurvity issues that may arise, using mobility data selected from Appendix section 1.4 and other independent variables. The significance level and the concurvity for each variable in every model are reported in table S4 below:

Appendix table S4: Significance level and concurvity for all dynamic variables.

|  | Pre-Delta wave | | Delta wave | | Omicron wave | |
| --- | --- | --- | --- | --- | --- | --- |
| Variable | Signif | Concurvity | Signif | Concurvity | Signif | Concurvity |
| Relative completed primary series rate | *** | 0.71 | *** | 0.79 | *** | 0.81 |
| Relative previous infection rate (12 weeks) | *** | 0.62 | *** | 0.31 | *** | 0.41 |
| Relative full-service restaurant visits |  | 0.82 |  | 0.81 | * | 0.86 |
| Relative gas station visits |  | 0.91 |  | 0.85 | ** | 0.89 |
| Relative religious organization visits | *** | 0.84 |  | 0.84 | ** | 0.87 |
| Relative gym visits | *** | 0.69 | ** | 0.76 |  | 0.76 |
| Relative university visits | * | 0.80 | *** | 0.62 | *** | 0.64 |
| Relative office of physician visits | *** | 0.70 |  | 0.65 | ** | 0.68 |
| Relative weekly testing rate | *** | 0.81 | *** | 0.64 | *** | 0.57 |
| Relative government response index | *** | 0.78 | *** | 0.32 |  | 0.27 |

Significance codes: ‘***’: 0.001, ‘**’: 0.01, ‘*’: 0.05, ‘.’: 0.1, ‘’: > 0.1.

The value of concurvity range from 0 to 1, the higher the concurvity the more a smooth variable can be approximated by the smooth of other variables. Specifically, a concurvity value above 0.8 generally signals the need for careful inspection of the model. Based on the results from table S4, we removed relative full-service restaurant, relative gas station, and relative religious organization visits from the model. This decision was based on their lack of significance and/or their high concurvity values. The equivalent results for selected variables are presented in table S5.

Appendix table S5: Significance level and concurvity for selected variables.

|  | Pre-Delta wave | | Delta wave | | Omicron wave | |
| --- | --- | --- | --- | --- | --- | --- |
| Variable | Signif | Concurvity | Signif | Concurvity | Signif | Concurvity |
| Relative completed primary series rate | *** | 0.63 | *** | 0.61 | *** | 0.60 |
| Relative previous infection rate (12 weeks) | *** | 0.59 | *** | 0.25 | *** | 0.35 |
| Relative gym visits | *** | 0.54 |  | 0.57 |  | 0.56 |
| Relative university visits |  | 0.69 | *** | 0.49 | *** | 0.44 |
| Relative office of physician visits | *** | 0.66 |  | 0.60 | ** | 0.64 |
| Relative weekly testing rate | *** | 0.78 | *** | 0.51 | *** | 0.51 |
| Relative government response index | *** | 0.72 | *** | 0.26 | * | 0.23 |

Significance codes: ‘***’: 0.001, ‘**’: 0.01, ‘*’: 0.05, ‘.’: 0.1, ‘’: > 0.1.

The results from table S5 reveal that each variable exhibits a concurvity value below 0.8 and is significant in at least one out of three models.

*2.3 Robustness check of vaccination data*

We selected the completed primary series rate as the main vaccination variable in the main analysis. To assess the robustness and validity of our findings, we conducted additional analyses using different vaccination data (completed primary series rate and partial vaccination rate) and varying starting dates (March 8^th^, 2021, and April 19^th^, 2021) for the analysis. We applied our sensitivity analysis to Model Pre-Delta-RCHR, as it is the only one that could be affected by the analysis. The results of four different combinations of vaccination data and starting date for Model Pre-Delta-RCHR are shown in Appendix figure S6 below:


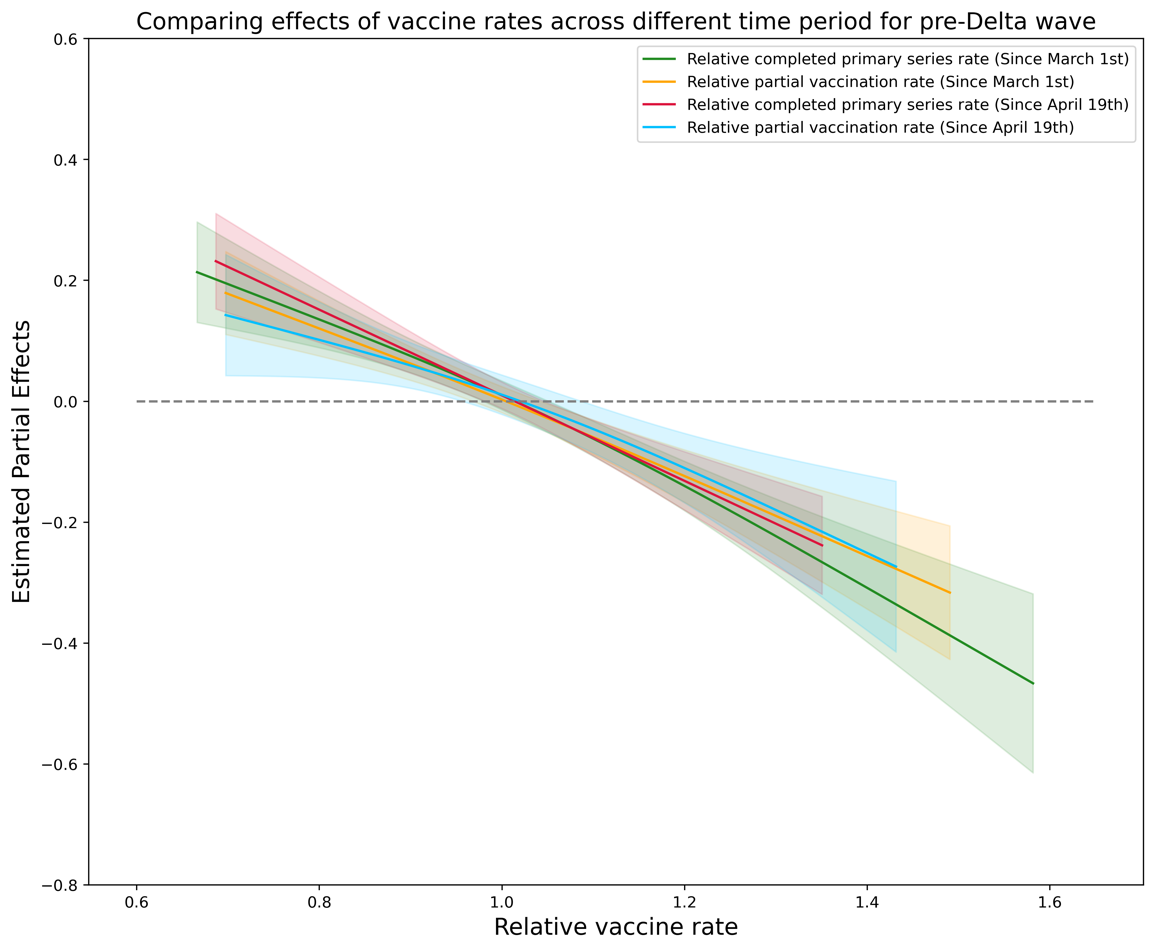


Appendix figure S6: Robustness check of vaccination rate with different vaccination data and varying starting date for Model Pre-Delta-RCHR.

The findings from this robustness check demonstrated a strong and consistent impact of vaccination, independent of the chosen vaccination data or the starting date of the analysis. This consistency suggests the robustness of our results and highlights the robustness of the completed primary series rate as the main vaccination variable.

*2.4 Sensitivity analysis of prior window length for previous infection*

In this section, we presented a sensitivity analysis to assess the impact of the prior window length for the previous infection on our analysis. To ensure our results are robust, we fixed all other covariates and a lag of four weeks for previous infections while varying the prior window length for previous infections from 12 to 24 weeks. The results of this sensitivity analysis for each model are shown below:


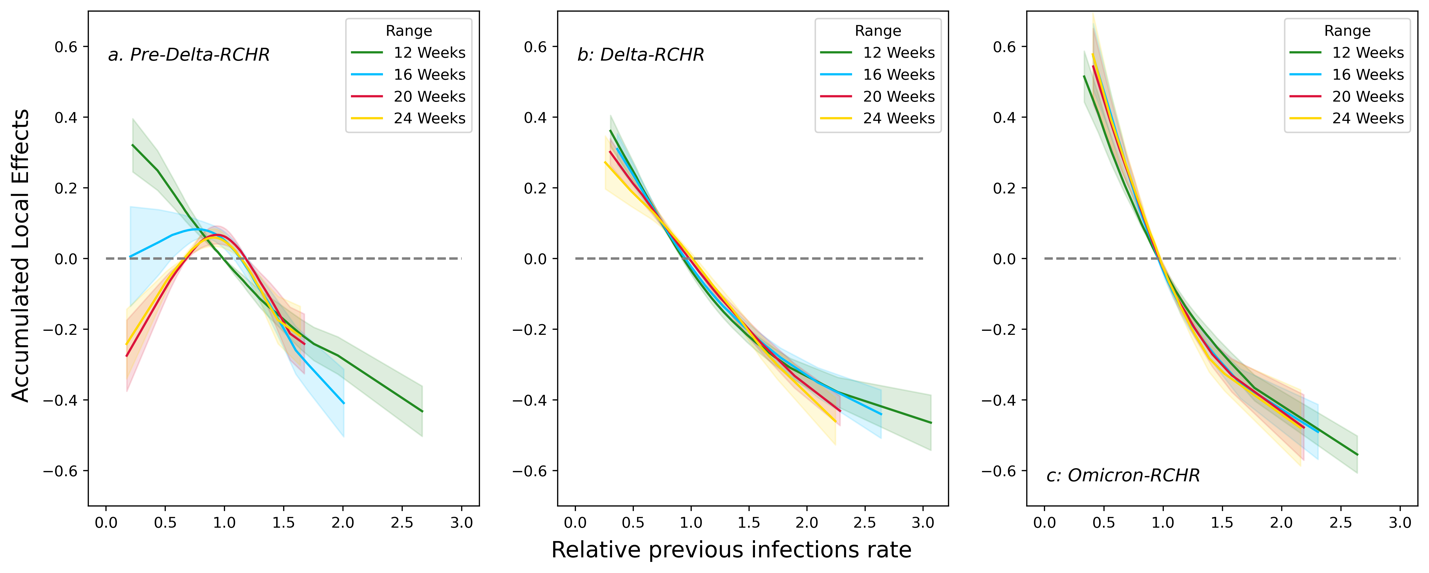


Appendix figure S7: Comparison of accumulated local effects of the previous infection rate for Model Pre-Delta-RCHR (a), Delta-RCHR (b), and Omicron-RCHR (c) with different prior window lengths (12, 16, 20 and 24 weeks).

*2.5 Sensitivity analysis of lags for previous infection*

This section presents a sensitivity analysis to assess the impact of the prior window length for the previous infection on our analysis. To ensure our results are robust, we fixed all other covariates and a prior window length of 12 weeks for previous infections while varying the lag for previous infections from 4 to 16 weeks. The results of this sensitivity analysis for each model are shown below:


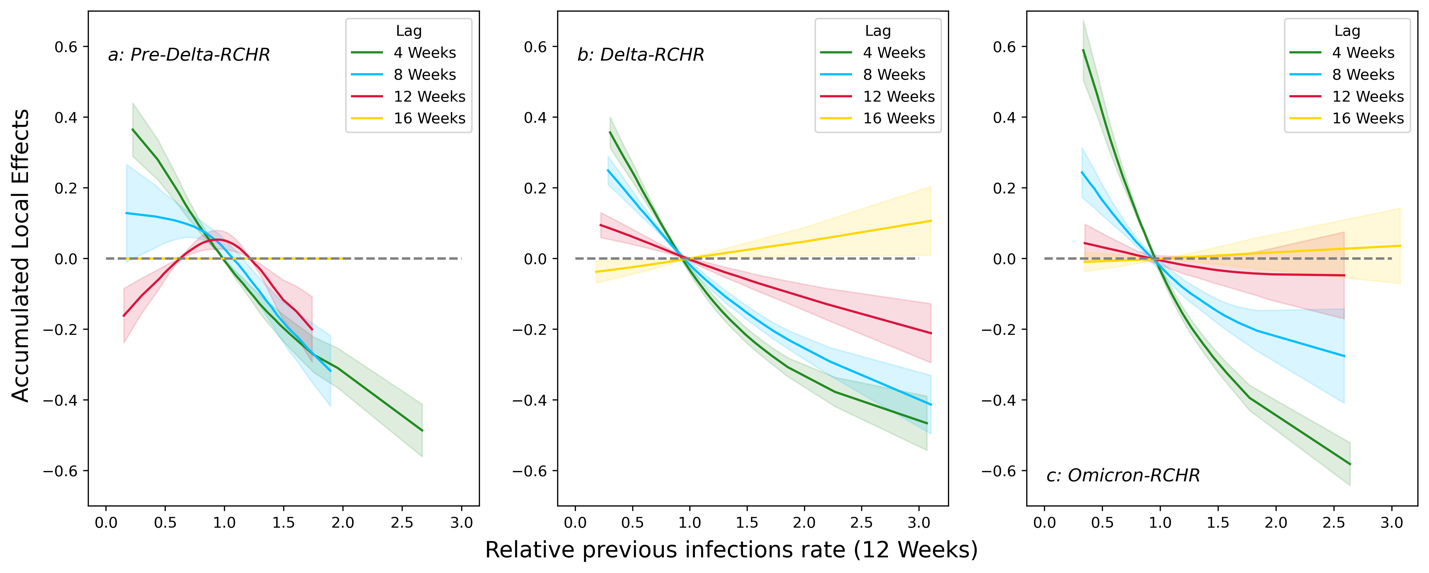


Appendix figure S8: Comparison of the accumulated local effects of the previous infection rate for Model Pre-Delta-RCHR (a), Delta-RCHR (b) and Omicron-RCHR (c) with different lags (4, 8, 12, and 16 weeks).

*2.6 GAMs with reported case-incidence rate (RCIR) as the outcome variable*

These GAMs share the same framework as Model Pre-Delta-RCHR, Delta-RCHR, and Omicron-RCHR, while the outcome variable is the reported case-incidence rate (RCIR). To account for the sequential process leading to infections, all lags between dynamic covariates and RCIR have been reduced by one week. These three GAMs have the form:

$${RCIR}_{i}^{t} \sim Gamma(\mu, \phi)$$

$$\log\left( \mu\right)= {\alpha+ f}_{1}(RCPSR_{i}^{t-1})+f_{2}(RWTR_{i}^{t-1})+ f_{3}\left( RGV_{i}^{t-1} \right)+ f_{4}\left( RPV_{i}^{t-1} \right)$$

$$f_{5}\left( RUV_{i}^{t-1} \right)+ f_{6}\left( RGP_{i}^{t-1} \right)+ f_{7}\left( {RPI}_{i}^{t} \right)+\beta_{1}\left( Black proportion \right)+ (S1)$$

$$\beta_{2}\left( SVI \right)+ \beta_{3}\left( Proportion of adults at high risk \right)+ \beta_{4}\left( Medicaid spending \right)$$

Where $\alpha$ represents the intercept, $\beta_{i}$ represent the parametric coefficients of each static variable, and $f_{i}$ are spline smooth functions of the relative dynamic variables. Additionally, a model is constructed for the Omicron wave, incorporating an interaction between completed primary series and booster rate (Omicron-Booster-RCIR). The model Omicron-Booster-RCIR has the form:

$${RCIR}_{i}^{t} \sim Gamma(\mu, \phi)$$

$$\log\left( \mu\right)= {\alpha+ f}_{1}(RCPSR_{i}^{t-1},RBR_{i}^{t-1})+f_{2}(RWTR_{i}^{t-1})+ f_{3}\left( RGV_{i}^{t-1} \right)+ f_{4}\left( RPV_{i}^{t-1} \right)$$

$$f_{5}\left( RUV_{i}^{t-1} \right)+ f_{6}\left( RGP_{i}^{t-1} \right)+ f_{7}\left( {RPI}_{i}^{t} \right)+\beta_{1}\left( Black proportion \right)+ (S2)$$

$$\beta_{2}\left( SVI \right)+ \beta_{3}\left( Proportion of adults at high risk \right)+ \beta_{4}\left( Medicaid spending \right)$$

Where $f_{1}$ represent a smooth interaction function between $RCPSR_{i}^{t-2}$ and $RBR_{i}^{t-2}$. For all the mentioned models above, the weekly state-level RCIR is assumed to follow a Gamma distribution with a log link. This choice of the Gamma family accounts for the positively skewed distribution of the outcome variable. We use thin plate regression splines as the smoothing basis for all $f_{i}$ and set the basis dimension to three to maximize the interpretability of the models.

**3. Supplementary Results**

*3.1 Models evaluation for GAMs with RCHR as outcome variable*


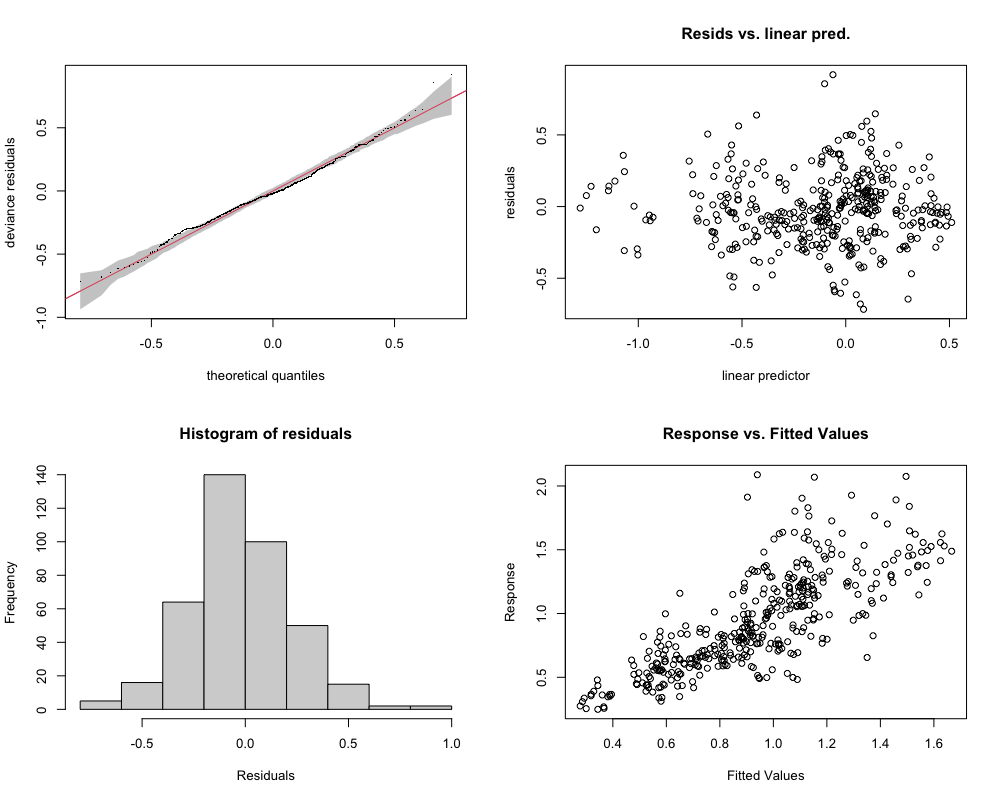


Appendix figure S9: Model diagnostic plots for Model Pre-Delta-RCHR. The correlation coefficient between fitted RCHR and predicted RCHR is 0.78.


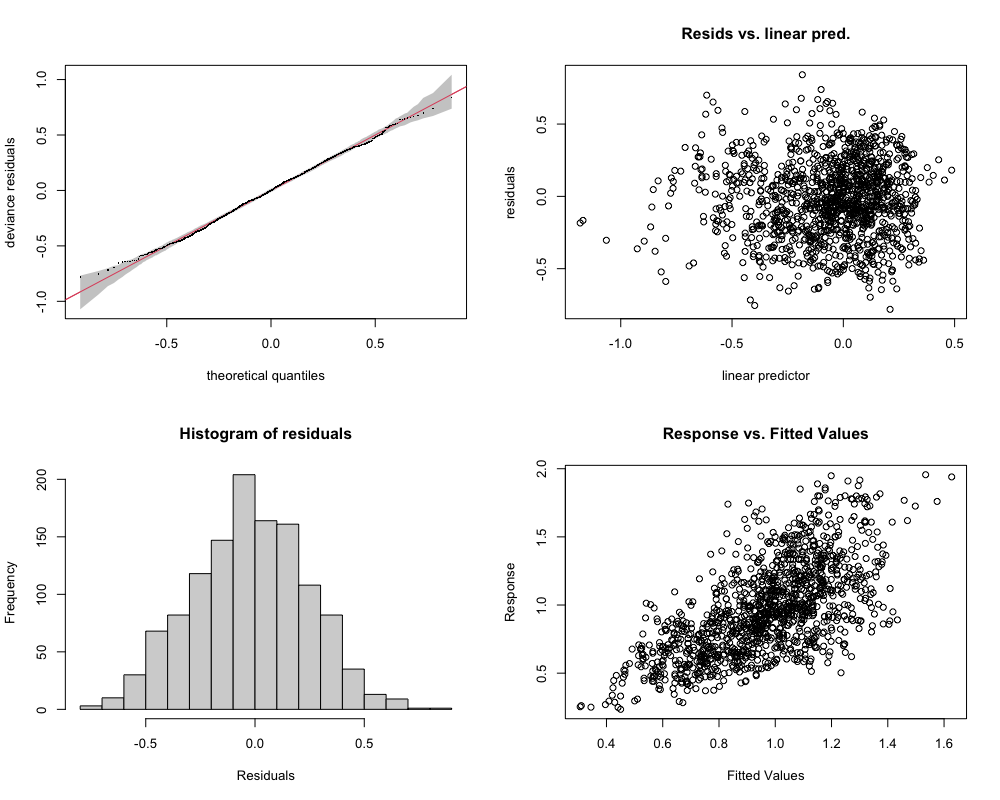


Appendix figure S10: Model diagnostic plots for Model Delta-RCHR. The correlation coefficient between fitted RCHR and predicted RCHR is 0.67.


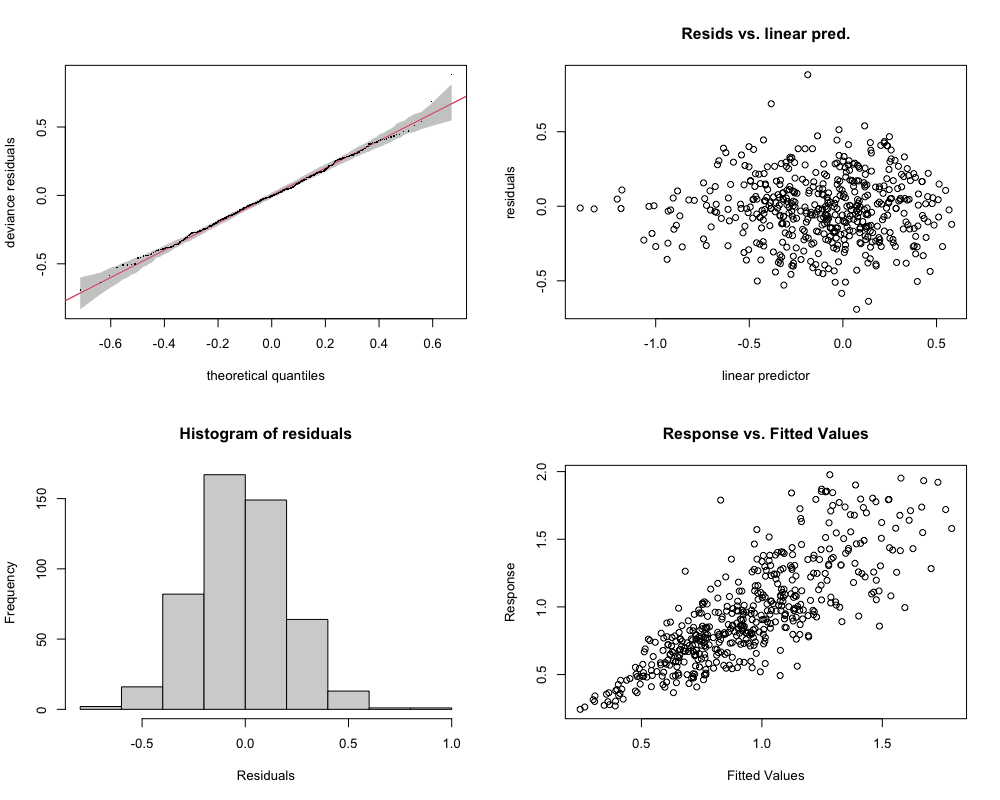


Appendix figure S11: Model diagnostic plots for Model Omicron-RCHR. The correlation coefficient between fitted RCHR and predicted RCHR is 0.81.


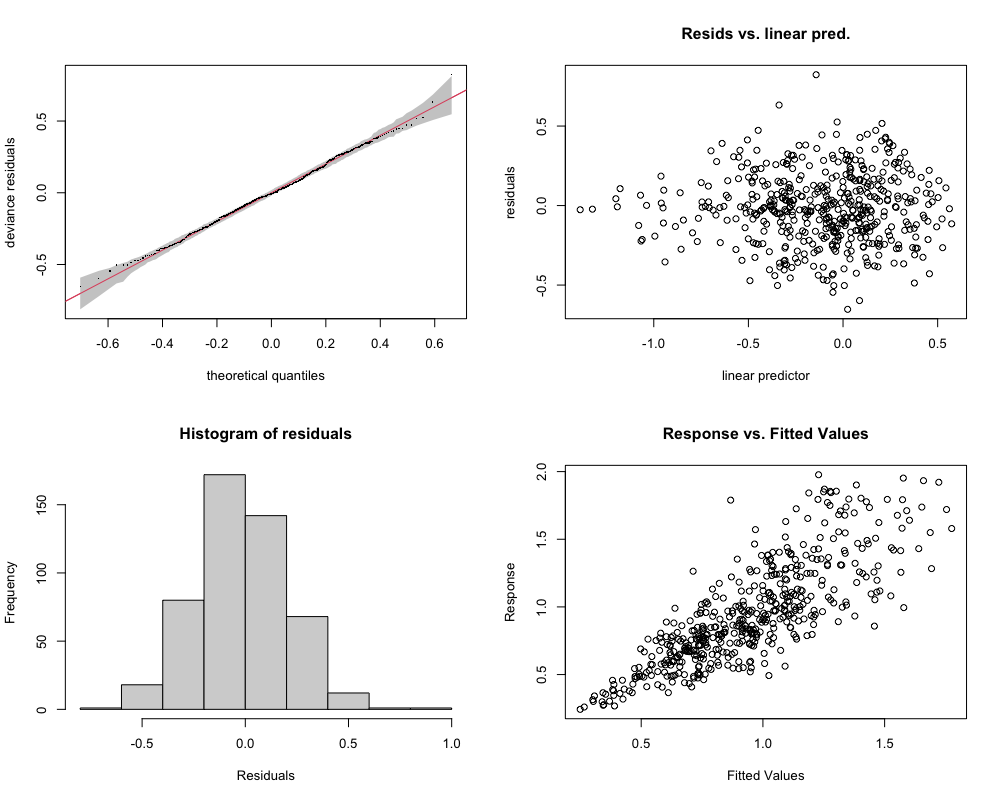


Appendix figure S12: Model diagnostic plots for Model Omicron-Booster-RCHR. The correlation coefficient between fitted RCHR and predicted RCHR is 0.83.

*3.2 Models evaluation for GAMs with RCIR as outcome variable*


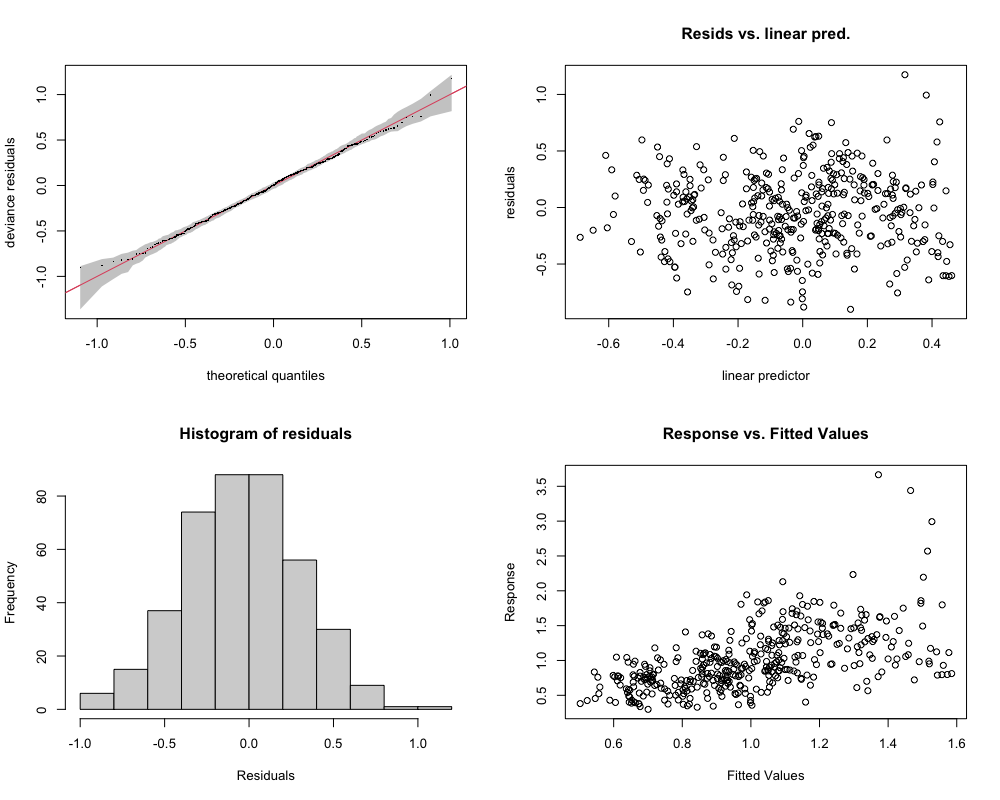


Appendix figure S13: Model diagnostic plots for Model Pre-Delta-RCIR. The correlation coefficient between fitted RCIR and predicted RCIR is 0.57.


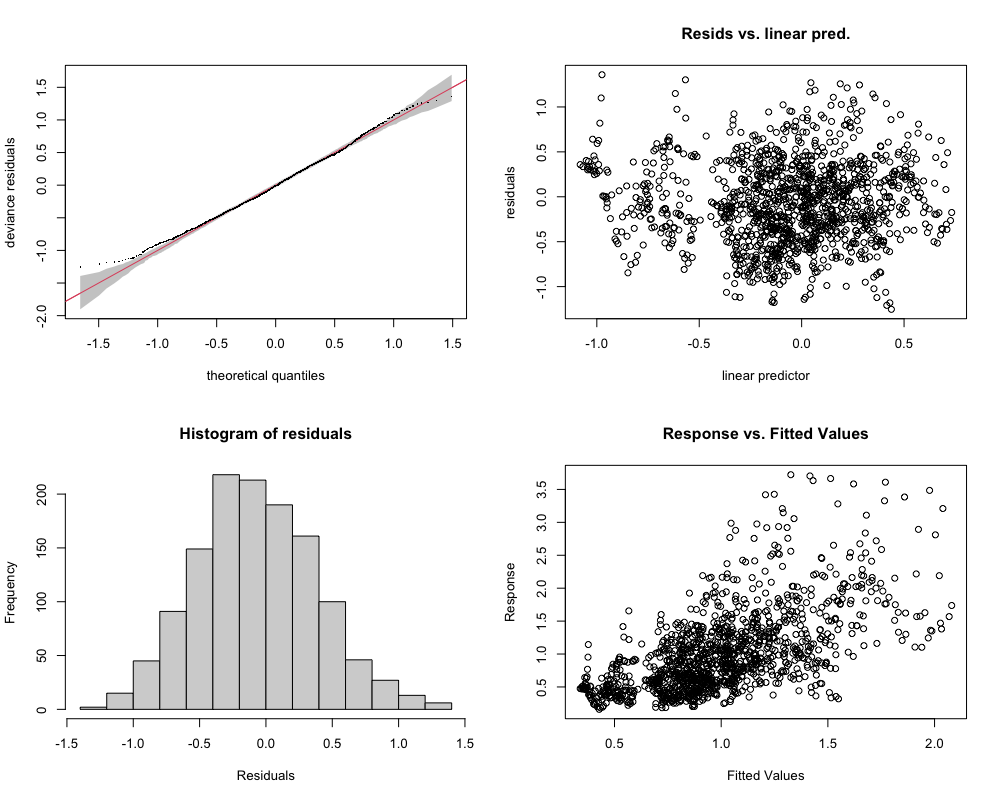


Appendix figure S14: Model diagnostic plots for Model Delta-RCIR. The correlation coefficient between fitted RCIR and predicted RCIR is 0.61.


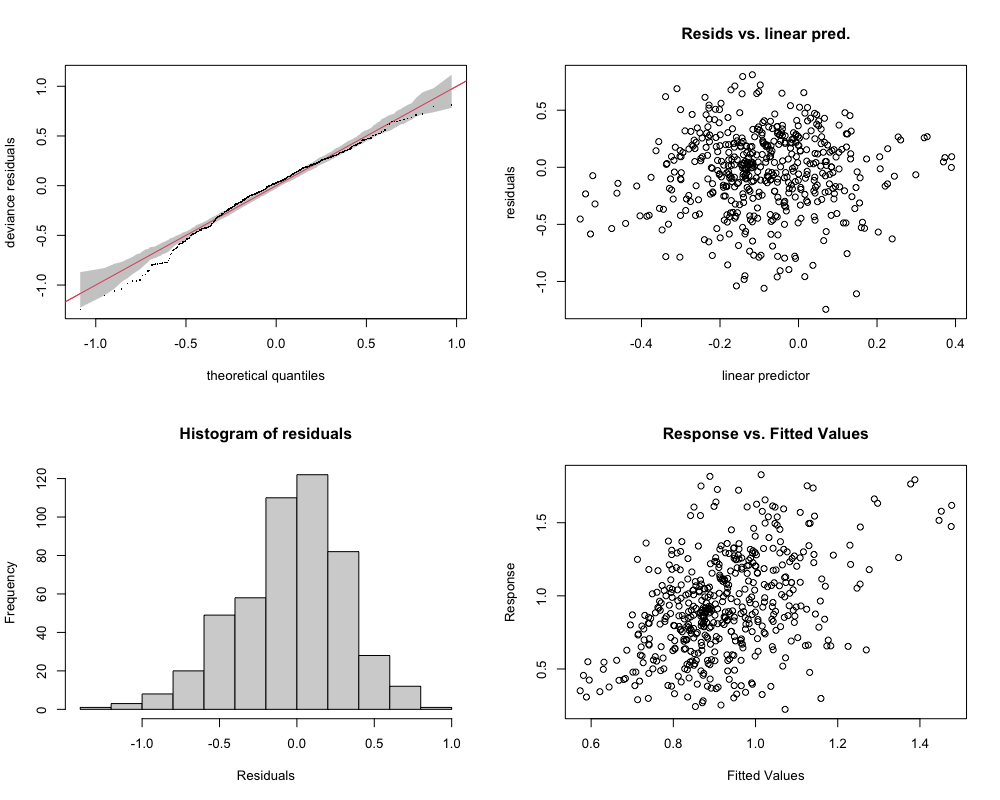


Appendix figure S15: Model diagnostic plots for Model Omicron-RCIR. The correlation coefficient between fitted RCIR and predicted RCIR is 0.43.


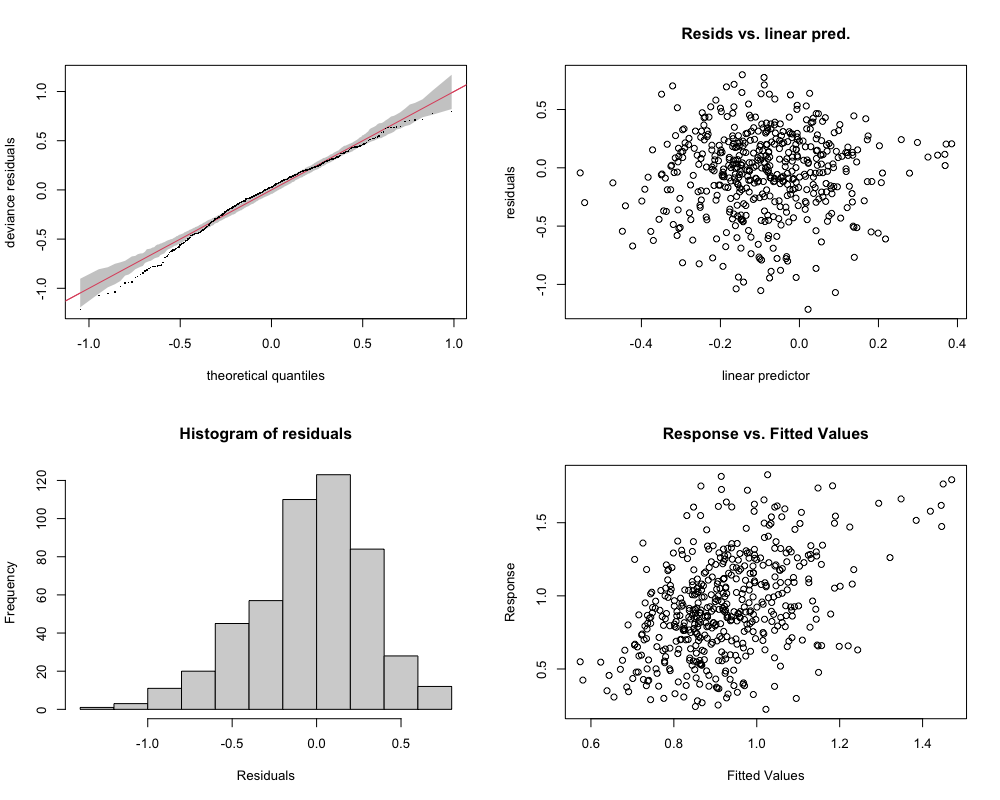


Appendix figure S16: Model diagnostic plots for Model Omicron-Booster-RCIR. The correlation coefficient between fitted RCIR and predicted RCIR is 0.44.

**Reference**

[1] Khare S, Gurry C, Freitas L, Schultz MB, Bach G, Diallo A, et al. GISAID’s role in pandemic response. China CDC Wkly 2021;3:1049.

[2] Faes C, Abrams S, Van Beckhoven D, Meyfroidt G, Vlieghe E, Hens N, et al. Time between symptom onset, hospitalisation and recovery or death: statistical analysis of Belgian COVID-19 patients. Int J Environ Res Public Health 2020;17:7560.

[3] Du H, Dong E, Badr HS, Petrone ME, Grubaugh ND, Gardner LM. Incorporating variant frequencies data into short-term forecasting for COVID-19 cases and deaths in the USA: a deep learning approach. eBioMedicine 2023;89:104482. https://doi.org/10.1016/j.ebiom.2023.104482.

[4] Places Data Curated for Accurate Geospatial Analytics | SafeGraph n.d. https://www.safegraph.com (accessed January 13, 2023).

[5] NAICS & SIC Identification Tools. NAICS Assoc n.d. https://www.naics.com/search/ (accessed April 6, 2023).

[6] Altarawneh HN, Chemaitelly H, Ayoub HH, Tang P, Hasan MR, Yassine HM, et al. Effects of Previous Infection and Vaccination on Symptomatic Omicron Infections. N Engl J Med 2022;387:21–34. https://doi.org/10.1056/NEJMoa2203965.

[7] Goldberg Y, Mandel M, Bar-On YM, Bodenheimer O, Freedman LS, Ash N, et al. Protection and Waning of Natural and Hybrid Immunity to SARS-CoV-2. N Engl J Med 2022;386:2201–12. https://doi.org/10.1056/NEJMoa2118946.

[8] Bobrovitz N, Ware H, Ma X, Li Z, Hosseini R, Cao C, et al. Protective effectiveness of previous SARS-CoV-2 infection and hybrid immunity against the omicron variant and severe disease: a systematic review and meta-regression. Lancet Infect Dis 2023;23:556–67. https://doi.org/10.1016/S1473-3099(22)00801-5.

[9] Abrahim SA, Tessema M, Defar A, Hussen A, Ejeta E, Demoz G, et al. Time to recovery and its predictors among adults hospitalized with COVID-19: A prospective cohort study in Ethiopia. PLOS ONE 2020;15:e0244269. https://doi.org/10.1371/journal.pone.0244269.

[10] Haug N, Geyrhofer L, Londei A, Dervic E, Desvars-Larrive A, Loreto V, et al. Ranking the effectiveness of worldwide COVID-19 government interventions. Nat Hum Behav 2020;4:1303–12.

[11] Hale T, Webster S, Petherick A, Phillips T, Kira B. Oxford COVID-19 government response tracker (OxCGRT). Last Updat 2020;8:30.

[12] Bureau UC. State Population by Characteristics: 2010-2019. CensusGov n.d. https://www.census.gov/data/datasets/time-series/demo/popest/2010s-state-detail.html (accessed April 9, 2023).

[13] Total Medicaid Spending. KFF 2022. https://www.kff.org/medicaid/state-indicator/total-medicaid-spending/ (accessed February 13, 2023).

[14] Health Care Expenditures by State of Residence (in millions). KFF 2022. https://www.kff.org/other/state-indicator/health-care-expenditures-by-state-of-residence-in-millions/ (accessed February 13, 2023).

[15] Poverty Rate by Race/Ethnicity. KFF 2022. https://www.kff.org/other/state-indicator/poverty-rate-by-raceethnicity/ (accessed February 13, 2023).

[16] CDC/ATSDR SVI Data and Documentation Download | Place and Health | ATSDR 2022. https://www.atsdr.cdc.gov/placeandhealth/svi/data_documentation_download.html (accessed February 13, 2023).

[17] Global Burden of Disease (GBD). Inst Health Metr Eval 2014. https://www.healthdata.org/gbd (accessed April 9, 2023).

[18] Harvard Dataverse. Harv Libr n.d. https://library.harvard.edu/services-tools/harvard-dataverse (accessed April 9, 2023).

[19] Adults at Higher Risk of Serious Illness if Infected with Coronavirus. KFF 2020. https://www.kff.org/other/state-indicator/adults-at-higher-risk-of-serious-illness-if-infected-with-coronavirus/ (accessed February 13, 2023).

[20] Zhang J, Dong X, Liu G, Gao Y. Risk and protective factors for COVID-19 morbidity, severity, and mortality. Clin Rev Allergy Immunol 2023;64:90–107.

[21] Johnson TF, Hordley LA, Greenwell MP, Evans LC. Associations between COVID-19 transmission rates, park use, and landscape structure. Sci Total Environ 2021;789:148123.

[22] Bollyky TJ, Castro E, Aravkin AY, Bhangdia K, Dalos J, Hulland EN, et al. Assessing COVID-19 pandemic policies and behaviours and their economic and educational trade-offs across US states from Jan 1, 2020, to July 31, 2022: an observational analysis. The Lancet 2023:S0140673623004610. https://doi.org/10.1016/S0140-6736(23)00461-0.
